# Linking oral microbiome with mental health: A review

**DOI:** 10.6026/973206300223667

**Published:** 2026-06-30

**Authors:** Maleha Maleha

**Affiliations:** 1 Researcher, Tact Force, Jacksonville, Florida, 32257, USA

**Keywords:** Oral microbiome, mental health, depression, anxiety, gut-brain axis, dental hygiene, microbial biomarkers, oral dysbiosis, Prevotella, Haemophilus, Rothia

## Abstract

The gut microbiome shapes depression and anxiety, yet the oral microbiome that seeds it daily remains overlooked. Therefore, it is of interest to review on the oral microbiome's role in the gut-brain axis. A reproducible signature emerges: enriched Prevotella with depleted Haemophilus and Rothia. Four pathways connect a dysbiotic mouth to the brain inflammation, bacterial translocation, neurotransmitter signalling and HPA-axis dysregulation. As adults see hygienists more than any other clinician, dental visits could screen, prevent and refer along this axis.

## Background:

Mental health disorders have become one of the defining public health challenges of our era, affecting more than 792 million people worldwide and accounting for roughly 13% of global disability-adjusted life years [1, 2]. Depression alone afflicts an estimated 264 million individuals and anxiety disorders affect a further 284 million. The economic burden is staggering-approximately US$2.5 trillion annually and projected to reach $6 trillion by 2030 [2]. Pharmacological treatment, while life-saving for many, remains imperfect: 30-40% of patients show inadequate response to first-line antidepressants [4]. This persistent treatment gap has fuelled interest in modifiable biological systems that might explain individual differences in vulnerability and response and the human microbiome has emerged as one of the most promising of these. That oral health and mental health travel together has been observed clinically for decades [4, 5]. Patients with depression neglect oral hygiene more often develop more periodontitis and experience worse treatment outcomes patterns long attributed to behavioural and motivational factors. What this review explores is the reverse and biologically more interesting possibility that disturbance of the oral microbial community themselves contribute upstream to mood and anxiety symptoms through measurable mechanisms [6, 7, 8]. The past two decades have witnessed a true microbiome revolution [9, 10, 11, 12]. The gut microbiome comprising trillions of microorganisms encoding more than three million unique genes (roughly 150 times the size of the human genome) now stands recognized as a critical regulator of systemic physiology through metabolite production, immune modulation (some 70% of immune cells reside in gut-associated tissue) and neuroendocrine signalling [13, 14]. The gut-brain axis represents bidirectional communication between the gastrointestinal microbiome and the central nervous system [9, 15], mediated by several overlapping pathways the vagus nerve, providing direct neural signalling; immune mediators including circulating cytokines and chemokines [16]; microbial metabolites such as short-chain fatty acids (butyrate, propionate, acetate) and tryptophan derivatives [17 18, 19] and neuroactive compounds including gamma-aminobutyric acid (GABA), serotonin and dopamine [17, 18]. Mast cells and microglia participate in this neuro-inflammatory crosstalk [20], while short-chain fatty acids influence the integrity of the blood-brain barrier [19, 20]. Notably, around 95% of the body's serotonin is produced in the gut [18], which underscores how intimately the microbial community shapes neurotransmitter homeostasis. Preclinical work in germ-free mice has shown that the absence of gut microbiota produces altered stress responses, abnormal neurotransmitter profiles in mood-relevant brain regions (prefrontal cortex, hippocampus, amygdala) and behavioural changes including heightened anxiety-like behaviour and reduced social interaction [21 22, 23]. Many of these deficits can be reversed through microbial colonization or administration of specific probiotic strains [24], which provides some of the strongest causal evidence for microbiome-brain interactions. Human clinical work has confirmed that patients with major depressive disorder (MDD) carry distinct gut microbial signatures, particularly altered Firmicutes-to-Bacteroidetes ratios [25 26, 27, 28, 29]. Therefore, it is of interest to describe the oral microbiome's role in the gut-brain axis and its implications for dental practice.

## The overlooked oral microbiome:

Whereas gut microbiome-mental health research has flourished, with more than 5,000 published studies [30, 31], the oral microbiome the second most diverse microbial ecosystem in the human body [32] has remained conspicuously absent from psychiatric investigation [6, 7]. The oral cavity harbours more than 700 identified species distributed across diverse ecological niches: Tongue dorsum, buccal mucosa, palate, tooth surfaces and saliva [32]. Unlike the gut, the oral cavity is easily accessible for non-invasive sampling [6], which makes it an unusually attractive site for both clinical assessment and research. Critically, the oral cavity functions as a continuous inoculation source for the gastrointestinal tract [33, 34]. Humans swallow roughly 1.5 litres of saliva each day, carrying between 10^s^ and 10^9^ bacteria per millilitre [32]. Strain-level tracking has confirmed that oral bacteria do indeed colonize the gut, with species such as Prevotella, Veillonella and Streptococcus routinely detected in faecal samples [33, 34]. This positions the mouth as an upstream determinant of gut composition rather than a separate microbial compartment and offers a tangible biological route by which oral dysbiosis could reach the gut-brain axis. From a dental hygiene perspective, the practical implication is striking. Adults typically see a dental hygienist two to four times a year for preventive care, often more frequently than they see their primary care physician [4]. If oral microbial alterations are linked to mental health disorders, then routine dental visits become a hitherto untapped opportunity for early detection, sampling and microbiome-aware intervention [4, 35].

## Why this question now:

Despite growing recognition of the gut-brain axis [9, 15] and increasing interest in oral-systemic health connections [36, 37], evidence specifically examining the oral microbiome in relation to mental health outcomes has not been brought together in a clinically usable way. Existing reviews have focused predominantly on gut microbial alterations in psychiatric conditions [30, 1, 31], while oral microbiome reviews have addressed cardiovascular disease, diabetes mellitus and Alzheimer's disease [38, 39 40]. Periodontal disease affects 47% of adults over the age of 30 in the United States, with severe periodontitis affecting 11% of adults globally [41]. Mental health disorders affect roughly 20% of adults each year [2]. The overlap is large enough that even modest causal contributions of oral dysbiosis to psychiatric symptoms or vice versa would carry meaningful population-level consequences. Recent reviews have correspondingly emphasized the urgency of synthesizing the oral-gut-brain axis in this context [30, 42].

## Microbiome assessment in the cited literature:

Across the body of work informing this review, oral microbiome samples were most often collected from saliva, with smaller proportions of studies using oral swabs or tongue dorsum samples. Nearly all groups employed next-generation sequencing and 16S rRNA gene sequencing - targeting the V3-V4 hypervariable region in the majority of studies-remained the dominant method, complemented in a minority of studies by shotgun metagenomics or metatranscriptomics. Illumina platforms (MiSeq, NovaSeq, HiSeq) accounted for the bulk of sequencing runs. This methodological convergence is strength: It allows reasonably direct comparison of taxonomic findings across populations, even where downstream analytic pipelines differ.

## Microbial composition and reproducible biomarkers:

## Beta diversity:

Studies that examined beta diversity the degree to which microbial communities differ between individuals have consistently found significant separation between people with and without mental health disorders, regardless of sequencing strategy or geographic setting. Mental health status appears to explain a real, if modest, fraction of the compositional variance in the oral microbial community.

## Alpha diversity:

By contrast, most studies have not detected significant alpha diversity differences that is, the within-sample richness or evenness of the oral microbial community is largely preserved in depression, anxiety and related conditions. This pattern suggests that mental health disorders are associated less with a wholesale impoverishment of the oral microbiome and more with compositional rebalancing among existing taxa.

## Reproducible biomarker taxa:

Three taxa have shown consistent associations across multiple independent studies and now stand out as candidate biomarkers for mental health risk stratification. Prevotella species, especially Prevotella nigrescens, are repeatedly enriched in depression. Haemophilus species, including Haemophilus parainfluenzae, are repeatedly depleted. Rothia species, particularly Rothia mucilaginosa, are likewise depleted. These three signals one upward and two downward forms a coherent and reproducible compositional fingerprint and a visual summary of the main results is presented in (Figure 1 - see PDF)

## Biological mechanisms linking the oral microbiome to mental health:

Animal and translational studies converge on four mechanistic pathways through which oral dysbiosis could plausibly contribute to mental health outcomes. These pathways are not mutually exclusive and most likely operate in concert.

## Inflammatory signalling:

Oral dysbiosis triggers elevated systemic levels of IL-6, TNF-α and IL-1 β-cytokines well established in the pathophysiology of depression. These inflammatory mediators can act on the brain through humoral routes and through effects on blood-brain barrier permeability, ultimately influencing neuronal function and mood regulation.

## Oral-to-gut bacterial translocation:

Strain-level analyses confirm that oral bacteria colonize the gut through the simple, continuous mechanism of saliva swallowing, establishing the oral cavity as an upstream determinant of gut microbial composition rather than a separate ecosystem. A dysbiotic mouth therefore feeds a dysbiotic gut and any gut-mediated effect on the brain inherits, at least in part, from the oral source community.

## Microbial modulation of neurotransmitters:

Oral bacteria possess enzymatic machinery to produce or modify GABA, serotonin precursors and dopamine metabolites. A dysbiotic oral community can also shift tryptophan metabolism away from serotonin synthesis and toward the neurotoxic kynurenine pathway, a metabolic detour repeatedly implicated in depression.

## HPA-axis dysregulation:

Chronic psychological stress induces oral dysbiosis and the resulting microbial shifts in turn exacerbate anxiety- and depression-like behaviour, creating a bidirectional loop in which mental distress and oral microbial disturbance reinforce one another. The four mechanistic pathways are illustrated in (Figure 2 - see PDF).

## The oral microbiome as a gateway to mental health:

Taken together, the body of work reviewed here makes a compelling case for the oral-gut-brain axis as a clinically meaningful framework. From a dental practice perspective, these findings reposition oral health professionals from peripheral observers to central players in mental health surveillance and early intervention. The consistency of beta diversity findings across diverse populations, age groups and geographic regions [43, 43, 44 45, 46, 47] suggests these associations are robust and generalizable rather than artefacts of any single cohort. Unlike the gut microbiome, which requires faecal sampling, the oral microbiome can be assessed through simple saliva collection or oral swabs [6], procedures that fit naturally into a dental hygiene appointment.

## Clinical implications for dental practice:

## The dental professional as mental health sentinel:

Dental hygienists and dentists hold structural advantages for mental health screening that remain largely untapped. Adults typically attend a dental hygiene appointment two to four times a year, often more frequently than they see their primary care physician, which provides repeated, longitudinal opportunities for assessment and early identification of at-risk individuals. Direct access to the oral cavity allows non-invasive sampling saliva collection or oral swabs are already routine and these samples could in time enable microbiome-based risk assessment as analytic technology becomes more affordable. Dental visits also tend to feel less stigmatizing than mental health appointments, which mean that dental professionals are in a position to identify individuals who would not otherwise seek psychological help. The therapeutic relationship developed during regular preventive care, often spanning many years, lends itself naturally to sensitive conversations about mood and stress. Practical implementation could include validated mental health screening tools during new patient examinations or periodic health-history updates. The Patient Health Questionnaire-2 (PHQ-2) offers a brief initial screen, with positive results prompting the full PHQ-9 for depression or the GAD-7 for anxiety. Visual examination for oral signs of psychological distress neglected hygiene, increased plaque accumulation, signs of bruxism, altered salivary flow provides additional clinical indicators. Combined with emerging microbiome-aware approaches, this dual strategy could identify patients in need of mental health referral as well as those who might benefit from oral microbiome-targeted preventive interventions.

## Therapeutic interventions within the dental scope of practice:

Several evidence-informed interventions sit comfortably within current dental hygiene practice and could plausibly contribute to better mental health outcomes through the oral-gut-brain axis.

## Enhanced mechanical plaque control:

Professional scaling and root planing, complemented by comprehensive education in effective home care proper brushing technique with soft-bristled brushes, interdental cleaning with floss or interdental brushes and tongue scraping reduces both bacterial load and inflammatory burden. Given the evidence for oral-to-gut translocation, thorough plaque control may reduce pathogenic bacterial seeding of the gut microbiome and thereby contribute to improved mental health outcomes through reduced systemic inflammation.

## Oral psychobiotics:

Emerging probiotic strains including Lactobacillus reuteri, Lactobacillus rhamnosus and Bifidobacterium longum appear to colonize both oral and gut environments. Animal studies have demonstrated that these strains can reduce anxiety- and depression-like behaviour and oral probiotic lozenges taken after brushing before bedtime offer a practical delivery route to maximize colonization. Dental professionals are well placed to recommend evidence-based probiotic strains as adjuncts to traditional periodontal therapy.

## Microbiome-aware dietary counselling:

Traditional caries-focused dietary advice can be expanded to include prebiotic fibres (inulin, fructooligosaccharides, resistant starches) that feed beneficial microbes and promote short-chain fatty acid production; omega-3 fatty acids with anti-inflammatory effects; polyphenol-rich foods (green tea, berries, dark chocolate) that modulate both oral and gut microbiomes; and a reduction in refined sugars, which favour dysbiotic shifts toward pathogenic species.

## Judicious antimicrobial use: 

Broad-spectrum antimicrobial mouth rinses (chlorhexidine, essential oils) reduce bacterial load effectively but also disrupt beneficial commensal microbes. In light of the findings reviewed here, dental professionals should reconsider indiscriminate antiseptic recommendation, reserving such agents for acute periodontal infections and otherwise favouring more selective approaches that preserve microbial diversity. Targeted antimicrobials specific to periodontal pathogens may be preferable for long-term use. The three-tier clinical implementation framework is presented in (Figure 3 - see PDF).

## Public health implications:

Given the high prevalence of periodontal disease (47% of adults over 30 in the United States) and mental health disorders (around 20% of adults annually), interventions targeting the oral-gut-brain axis could deliver substantial population-level benefit. Integrating mental health screening into dental visits could identify undiagnosed cases through a non-stigmatizing healthcare touch point, reduce disparities by reaching underserved populations who access dental care but not mental health services and provide low-cost interventions that complement rather than replace standard psychiatric treatment.

## Conclusion:

The oral microbiome is no bystander to mental health but an accessible, modifiable partner in the gut-brain axis. Reproducible shifts in Prevotella, Haemophilus and Rothia, with four converging mechanisms, give it real biomarker and therapeutic value. Dental practice is uniquely placed to act on this axis, making its translation a priority for the coming decade.

## Funding:

This work received no external funding.

## Ethical approval:

This narrative review analysed previously published data and did not require IRB approval.

## Institutional oversight:

Not applicable.

## Pre-print status:

This manuscript has not been posted as a pre-print on any platform (e.g., bioRxiv, medRxiv, ResearchGate).

## References

[1] https://www.who.int/.

[2] https://pubmed.ncbi.nlm.nih.gov/35026139/.

[3] Rush AJ (2006). Am J Psychiatry..

[4] Cryan JF (2019). Physiol Rev..

[5] Sender R (2016). PLoS Biol..

[6] Foster JA (2017). Neurobiol Stress..

[7] Dewhirst FE (2010). J Bacteriol..

[8] Wingfield B (2021). Sci Rep..

[9] Li C (2022). Front Psychiatry..

[10] Simpson CA (2020). Physiol Behav..

[11] Malan-Müller S (2024). Transl Psychiatry..

[12] Paudel D (2022). Jpn Dent Sci Rev..

[13] Borrego-Ruiz A, Borrego JJ. (2025). AIMS Microbiol..

[14] Scassellati C (2021). Front Psychiatry..

[15] Bowland GB, Weyrich LS. (2022). Front Psychiatry..

[16] Martínez M (2022). Front Psychiatry..

[17] Dinan TG, Cryan JF. (2017). Gastroenterol Clin North Am..

[18] Kelly JR (2017). Front Neurosci..

[19] Sampson TR, Mazmanian SK. (2015). Cell Host Microbe..

[20] Belkaid Y, Hand TW. (2014). Cell..

[21] Dantzer R (2008). Nat Rev Neurosci..

[22] Strandwitz P. (2018). Brain Res..

[23] O'Mahony SM (2015). Behav Brain Res..

[24] Petra AI (2015). Clin Ther..

[25] Eke PI (2015). J Periodontol..

[26] Könönen E (2019). J Clin Med..

[27] Hajishengallis G, Chavakis T. (2021). Nat Rev Immunol..

[28] Silva YP (2020). Front Endocrinol..

[29] Venegas DP (2019). Front Immunol..

[30] Naseribafrouei A (2014). Neurogastroenterol Motil..

[31] Jiang H (2015). Brain Behav Immun..

[32] Aizawa E (2016). J Affect Disord..

[33] Kelly JR (2016). J Psychiatr Res..

[34] Lin P (2017). J Affect Disord..

[35] Yong SJ (2020). Front Neurosci..

[36] Simpson CA (2021). Clin Psychol Rev..

[37] Nikolova VL (2021). JAMA Psychiatry..

[38] Dominy SS (2019). Sci Adv..

[39] Ide M (2016). PLoS ONE..

[40] Ball J, Darby I. (2022). Periodontol 2000..

[41] Zheng DX (2021). J Clin Periodontol..

[42] Kitamoto S (2020). Cell..

[43] Atarashi K (2017). Science..

[44] Narengaowa (2021). Front Cell Neurosci..

[45] Lozupone CA (2012). Nature..

[46] Ursell LK (2012). Nutr Rev..

[47] https://pubmed.ncbi.nlm.nih.gov/22699609/.

